# Editorial: Bone cell differentiation in health and disease, volume II

**DOI:** 10.3389/fendo.2024.1499544

**Published:** 2024-11-22

**Authors:** Chandi C. Mandal, Julie A. Rhoades

**Affiliations:** ^1^ Department of Biochemistry, School of Life Sciences, Central University of Rajasthan, Ajmer, India; ^2^ Tennessee Valley Healthcare System, Department of Veterans Affairs, Nashville, TN, United States; ^3^ Department of Medicine, Vanderbilt University Medical Center, Nashville, TN, United States

**Keywords:** bone cell differentiation, bone health, bone disease, actin binding Plastin 3, x-box binding protein 1, short-chain fatty acids

Bone health relies on maintaining the balance between resorption and formation of the bone by the coordinating action of osteoclast and osteoblast cells. This intricate process of bone homeostasis is essential for preserving the bone’s quality and structural integrity. Osteoblast cells that induce bone matrix formation and mineral deposition, belong to the mesenchymal stem cell lineage. In contrast, osteoclasts work by causing the resorption of the mineralized matrix and belong to hematopoietic lineage. In addition to the osteoblast and osteoclast, osteocytes, immune cells and other cell types present in the bone and bone marrow coordinate with the endocrine system to establish and maintain a healthy skeleton. An imbalance in bone metabolism leads to of changes in bone structure and microarchitecture that cause bone fragility and fracture (1). In addition, to metabolic disease that result in imbalance bone homeostasis, tumors and cancer therapies can alter the communication between cells in the bone/bone marrow microenvironment leading to a similar imbalance in bone homeostasis. Specifically, tumor cells can promote osteoclast activity to develop osteolytic bone metastasis or induces abnormal osteoblast function to create osteoblastic bone metastasis in various cancer types including breast and prostate cancers (2, 3). This special topic in “*Bone Cell Differentiation in Health and Disease, Volume II*” presents papers that describe the crucial role of various genes like PLS3, XBP1 and GNAS, and short chain fatty acids producing foods in regulating bone metabolism and bone health, and subsequent mechanism of fibroblasts associated bone quality. 

## Dysregulation of various genes on bone diseases

Actin binding Plastin 3 (PLS3) protein regulates several cellular processes such as DNA repair, actin dynamics, membrane trafficking and endocytosis. However, abnormality in PLS3 protein is associated with various bone diseases including osteoporosis, osteoarthritis and osteogenesis imperfecta (brittle bone disease). Zhong et al., observed malformed body, muscle damage, and craniofacial abnormalities in zebrafish upon knockdown of PLS3 gene. The presence of PLS3 in the osteocytes supports the cytoskeletal integrity and regulates signals to mechanical stimuli by oscillating Ca^2+^ signalling. In the case of mechanical loading, osteoblasts tend to respond by inducing bone remodelling via cytoskeletal alterations. Function of osteoclast cells is also modulated by PLS3 via the NFκB signalling pathway. It interacts with NFκB repressing factor to inhibit the activation of master regulator NFATC1to modulate osteoclast activity.

The study by Lv et al., summarized that osteoblast cells produce extracellular matrix (ECM) proteins during skeletal development process, and it also exerts endoplasmic reticulum stress upon activation of unfolded protein response (UPR). To restore cellular homeostasis, unfolded protein response gets activated, and this ER stress is sensed by three key regulator proteins like ATF6, IRE1α and PERK. x-box binding protein 1 (XBP1) regulates endoplasmic reticulum homeostasis. Upon silencing of the IRE1α/XBP1 cascade in murine bone marrow macrophages, it was observed that osteoclast formation was reduced. This axis also modulates the PTH/PTHrP pathway stimulating osteoclastogenesis via RANKL. XBP1 directly binds to the promoter of osteoblast transcription factor osterix to regulate its expression inducing osteoblast differentiation. It also binds to NFATc1 promoter to enhance osteoclast activity. Similarly, XBP1 also induces pro-inflammatory cytokines in immune cells in response to annexin 2, TLR and NFκB signalling to upregulate osteoclast activity. However, in response to estrogen, this protein promotes anti-inflammatory signals to promote osteoblast differentiation. Thus, similar to PLS3, XBP1 protein regulates bone metabolism by regulating activity of osteoclast, osteoblast and immune cells.

GNAS dysregulation affects bone metabolism especially on regulating of PTH/PTHrP signalling pathway via Gsα-cAMP pathway. Heterotopic ossification (HO) is the dysregulation of cell fate determination induced by the percussor cells differentiating into bone, often due to GNAS mutations. In a study by Elli et al., the silencing of GNAS caused a premature transition between osteoblast to osteocytes along with elevated expression of an early marker for young osteocytes i.e., DMP1. The silencing of GNAS also causes unusual matrix deposition leading to ectopic bone formation by upregulating osteoblast transcription factor RunX2. Future research should focus on in-depth understanding the molecular cascades triggered by PLS3, XBP1 and GNAS to regulate bone metabolism to explore potential clinical applications.

## Mechanism of Fibroblasts regulated bone health

Wang et al., have addressed the existence of a complex interaction among different types of bone resident cells (osteoclasts, osteoblasts and osteocytes) and bone marrow stromal cells (e.g., fibroblasts, endothelial and immune cells) via various cytokines, chemokines and growth factors. This complex interaction leads to develop fracture healing by three sequential events (e.g., inflammation, repair, and remodelling). Here, fibroblast cells play a crucial role in fracture healing and bone remodelling by modulating the functions other cells like osteoblast, chondrocyte, osteocyte, osteoclast and endothelial cells. They can undergo osteogenesis during the fracture healing process with the help of various cytokines and growth factors such as FGFs, TGF-β, BMPs, and IGF-1. IGFBP7 induces trans-differentiation of fibroblasts into functional osteoblasts by phenotypic switching via IL-6-dependent pathway. Signalling pathways like the IGF-1/PI3K/Akt pathway can be modulated to induce fibroblasts to differentiate into functional osteoblasts, offering the potential for improved fracture healing and targeted therapies.

## Short-chain fatty acids link between bone turnover and food consumption

The study by Behler-Janbeck et al., suggests that the type of food consumption and simultaneous action of gut microbiota may have an impact on the bone turnover of an individual. When the gut microbes ferment compounds like inulin which are non-digestible, they produce certain short-chain fatty acids (SCFAs) that lead to a decline in bone turnover. The SCFAs are known to activate the G protein-coupled receptor (Gpr) 41 and 43. Double knockout mouse for Gpr41/43 fed with chow and inulin showed an increased trabecular bone volume. Acetate, one of the SCFAs, was given to differentiated osteoblasts isolated from bone marrow, resulting in reduced mineralization and the downregulation of key bone formation markers like Col1a1, Phex, and Ptgs2. The bone architecture is influenced by the SCFAs acting via the Gpr41/43 axis regulating the differentiation of mesenchymal cells to osteogenic fate.

Indeed, this notable Research Topic opens a new vision on the mechanism of bone metabolism and homeostasis which will be monitored by basic and clinical studies to develop therapy in future.
